# Associations of Internet Website Use With Weight Change in a Long-term Weight Loss Maintenance Program

**DOI:** 10.2196/jmir.1504

**Published:** 2010-07-27

**Authors:** Kristine L Funk, Victor J Stevens, Lawrence J Appel, Alan Bauck, Phillip J Brantley, Catherine M Champagne, Janelle Coughlin, Arlene T Dalcin, Jean Harvey-Berino, Jack F Hollis, Gerald J Jerome, Betty M Kennedy, Lillian F Lien, Valerie H Myers, Carmen Samuel-Hodge, Laura P Svetkey, William M Vollmer

**Affiliations:** ^6^Duke University Medical CenterDurhamUSA; ^5^Towson University, Department of KinesiologyTowsonUSA; ^4^University of VermontBurlingtonUSA; ^3^Pennington Biomedical Research CenterBaton RougeUSA; ^2^Johns Hopkins University School of MedicineBaltimoreUSA; ^1^Kaiser Permanente, Center for Health ResearchPortlandUSA

**Keywords:** Weight maintenance, Internet, intervention, weight loss, behavioral strategies

## Abstract

**Background:**

The Weight Loss Maintenance Trial (WLM) compared two long-term weight-maintenance interventions, a personal contact arm and an Internet arm, with a no-treatment control after an initial six-month Phase I weight loss program. The Internet arm focused on use of an interactive website for support of long-term weight maintenance. There is limited information about patterns of website use and specific components of an interactive website that might help promote maintenance of weight loss.

**Objective:**

This paper presents a secondary analysis of the subset of participants in the Internet arm and focuses on website use patterns and features associated with long-term weight maintenance.

**Methods:**

Adults at risk for cardiovascular disease (CVD) who lost at least 4 kilograms in an initial 20-week group-based, behavioral weight-loss program were trained to use an interactive website for weight loss maintenance. Of the 348 participants, 37% were male and 38% were African American. Mean weight loss was 8.6 kilograms. Participants were encouraged to log in at least weekly and enter a current weight for the 30-month study period. The website contained features that encouraged setting short-term goals, creating action plans, and reinforcing self-management habits. The website also included motivational modules, daily tips, and tailored messages. Based on log-in and weight-entry frequency, we divided participants into three website use categories: consistent, some, and minimal.

**Results:**

Participants in the consistent user group (n = 212) were more likely to be older (*P* = .002), other than African American (*P* = .02), and more educated (*P* = .01). While there was no significant difference between website use categories in the amount of Phase I change in body weight (*P* = .45) or income (*P* = .78), minimal website users (n = 75) were significantly more likely to have attended fewer Phase I sessions (*P* = .001) and had a higher initial body mass index (BMI) (*P* < .001). After adjusting for baseline characteristics including initial BMI, variables most associated with less weight regain included: number of log-ins (*P* = .001), minutes on the website (*P* < .001), number of weight entries (*P* = .002), number of exercise entries (*P* < .001), and sessions with additional use of website features after weight entry (*P* = .002).

**Conclusion:**

Participants defined as consistent website users of an interactive behavioral website designed to promote maintenance of weight loss were more successful at maintaining long-term weight loss.

**Trial Registration:**

NCT00054925; http://clinicaltrials.gov/ct2/show/NCT00054925 (Archived by WebCite at http://www.webcitation.org/5rC7523ue)

## Introduction

Obesity is a significant public health problem [1]. Behavioral interventions are standard treatment for encouraging healthy weight loss [2]. Unfortunately, behavioral interventions are intensive, and access and adherence to them typically limited. A majority of American adults (74%) have used the Internet [3]. Among adults aged 50 to 64, 44% reported using the Internet as a source of fitness and exercise information, while 31% reported looking online for information about weight control [4]. These numbers and other recent studies suggest that the Internet could be a vehicle for delivering behavioral weight-loss treatments [5-7].

Despite a proliferation of Internet weight-loss programs, detailed information is lacking about which Web components are necessary to promote adherence and long-term success. Limited information has suggested that log-in frequency and use of interactive features are related to weight loss [8,9]. Hurling et al [10] compared the effect of a highly interactive website encouraging physical activity with one that was less interactive; participants using the more interactive website were more likely to continue logging in and logged in more frequently over the study period. However, associations between various website features and outcomes were not evaluated. We and other researchers have postulated that increasing frequency of website utilization and duration of website use are likely to improve weight loss and maintenance. Therefore, the development of attractive and engaging Internet-based weight-control programs should be a priority. However, to maximize effectiveness and minimize cost it is important to assess which components, if any, of a website encourage long-term engagement and best assist with weight-loss maintenance.

The Weight Loss Maintenance Trial (WLM) [11], one of the longest running and largest weight-maintenance trials to date, was designed to test the efficacy of different long-term intervention strategies for helping participants maintain weight loss. One of the treatment conditions, the Internet arm, involved an interactive website that encouraged self-management skills for weight maintenance that were established during an initial six months of face-to-face weekly group meetings. The Internet intervention enrolled 348 participants and presented a unique opportunity to evaluate the specific elements of website utilization, both patterns and features, in an intervention designed to maintain weight loss. Therefore, the aims of this investigation were to (1) describe participant website-use patterns and (2) evaluate which Web features were associated with weight-loss maintenance.

## Methods

Sponsored by the National Heart, Lung, and Blood Institute (NHLBI), WLM was a 30-month, 4-center, randomized clinical trial testing the long-term efficacy of different strategies for maintaining weight loss. The study was approved by the institutional review board at each participating site and by an NHLBI-appointed protocol-review committee. All participants provided written informed consent.

In Phase I of the study, all participants enrolled in a six-month initial weight-loss program focusing on reducing caloric intake and increasing moderate-intensity physical activity [12]. Since weight loss of 4 kilograms (kg) is considered clinically significant for maintaining a health benefit [13,14], in Phase II, participants who lost 4 kg or more were randomly assigned to one of three maintenance conditions: a no-treatment self-directed control, a personal contact maintenance program involving monthly interaction with a health counselor, or an Internet maintenance program. The mean weight change (regain) at 30 months for each of the three conditions was 5.5 kg in the self-directed control group, 5.2 kg in the Internet maintenance program, and 4.0 kg in the personal contact maintenance program. The WLM study design and final results are published elsewhere [11,15]. This paper focuses on those randomized into the Internet condition (N = 348). The Internet arm involved use of an interactive website designed to facilitate and encourage behavioral skills initiated during Phase I and to aid in long-term weight-loss maintenance. The weight data used for final outcome analysis was collected in person at clinic visits for all three maintenance conditions. The final data-collection window began at 28 months. The study ended for an individual once his or her final weight had been collected. Therefore, to be consistent when comparing use of website features, this analysis compares website data for each Internet participant’s first 28 months of use.

### Participant Selection

Each participating institution recruited adults with a body mass index (BMI = weight in kg divided by height in m^2^) of 25 or greater through 45 who were taking medication for either hypertension or hyperlipidemia. To be eligible, volunteers were required to demonstrate Internet and email access by responding to an email and logging on to a screening website.

### The Internet Intervention

Participants randomized to the Internet group used an interactive website to self-monitor weight and enter information about diet, physical activity, and other weight-loss activities (eg, setting goals, making action plans, and getting support). The website provided unlimited access for 30 months to important behavioral intervention elements including a bulletin board, record-keeping tools, goal-setting modules, diet and exercise information, and tailored feedback. A description of the website design and implementation process has been published [16] as has the cost of developing the website [17]. The website was designed to support weight-loss maintenance using 5 key behavioral strategies: (1) reinforcing existing behavioral self-management, (2) encouraging new self-management skills, (3) improving self-efficacy, (4) encouraging long-term use of the website through providing innovative content, and (5) promoting social support.

Just after randomization, participants in the Internet group were oriented to the website at a one-time individual meeting with a weight-loss counselor. At the 45- to 60-minute orientation, participants set up a user account and established personal goals and plans for maintaining weight loss. They were instructed to log on to the website on their own within two days of the orientation and at least weekly for the duration of the 30-month study. At the orientation visit, the counselors encouraged participants to continue using specific lifestyle modifications similar to those used in Phase I of the study, including limiting calories, engaging in regular moderate-intensity exercise (goal of 225 minutes per week), and keeping food records. These targets were reiterated throughout the website.

The website contained multiple interactive pathways intended to support participants’ weight-management efforts, similar to in person group meetings. For example, just as participants might weigh in at the beginning of an in person group session, upon log-in, participants were immediately directed to the weight-entry screen. Participants could also check in by providing weekly progress updates (eg, enter weight, food records, and exercise minutes and select a date to return to the website). They could also revise goals, view their weight graph, and see a summary of progress compared with goals. Additional website features included a bulletin board for group interaction and discussion; a motivation center that provided tailored responses based on participants’ answers to inquiries about progress; an information center that contained reliable resources related to diet and physical activity; and a homepage “hub” that displayed participant profiles, a weekly interactive poll, and tailored checklists with suggested website activities.

We hypothesized that using the website consistently to track weight was an important component of successful weight-loss maintenance. To encourage this self-monitoring tool, the website was programmed to require entry of weight at least weekly. The website calculated the number of days since the participant’s last weight entry at each log-in; if more than seven days had elapsed since a weight entry, all areas of the website were disabled for that participant until a weight was entered. Once a weight was entered, links to the other features of the website were enabled. A series of automated email prompts and reminders encouraged regular log-ins. Participants who missed a weekly log-in date were sent an automated email reminder that was repeated after another week of no contact. After two weeks of no contact, participants received two weekly, automated telephone calls. If this did not result in a log-in, study staff contacted participants by telephone to encourage returning to the website; no behavioral weight-loss counseling was provided over the phone.

### Website-use Categories

For this analysis, we categorized participants into three groups based on number of log-ins and number of weight entries. While participants were asked to visit the website weekly, our operational definition of “consistent use” was logging in and having at least one weight entry every month for 26 of the 28 study months. We defined “some use” as logging in and having at least one weight entry in 14 to 25 of the 28 study months. All other use was defined as “minimal use.” We also defined the number of times a specific website feature was accessed for each of the participant-use categories. Website-use features analyzed were those related to behavioral self-management, including entry of weight and exercise, use of a social support component (ie, a bulletin board) and total time spent on the website. We defined one variable, “sessions with additional use after weight entry,” to capture the number of sessions in which participants used the website beyond the weekly “required” weight entry. This included bulletin-board use as well as the numerous features accessible to participants once they had entered a weekly weight.

### Weight Outcomes

We present several different weight outcome measures: (1) Phase II weight change (regain), (2) proportion of initial weight loss regained, and (3) proportion of participants at least 4 kg below initial weight. Final weights were collected for 323 of 348 (93%) Internet participants.

### Statistical Methods

All analyses were conducted using SAS, version 9.1 (SAS Institute Inc, Cary, NC, USA). Tests at *P* < .05 were considered significant. Multiple imputation techniques to account for missing data are described elsewhere [15]. We used logistic and multiple linear regression analyses, as appropriate, to adjust selected weight outcomes for race, sex, education, income, age, and initial BMI. Suitably weighted point estimates, standard deviations, and *P* values were calculated using the MIANALYZE procedure in SAS.

## Results

### Participants and Website Use

Over 65% of the 348 Internet participants were still actively logging on to the website at 28 months after randomization. Table 1 shows characteristics for all individuals in the Internet group and each of the three subgroup categories.

Table 2 shows overall use of website features during the first 28 months for all Internet participants. In general, 50% of participants logged on at least 107 times and spent over 400 minutes on the website. Sessions lasted 3 to 5 minutes on average. During roughly 80% of log-ins, participants used website features in addition to the required weight entry. Participants read more bulletin board messages than they posted.

**Table 1:** Characteristics for all Internet participants and each subgroup category

	All (N = 348)	Consistent (n = 212)	Some (n = 61)	Minimal (n = 75)	*P* value^a^
Male	37%	42%	31%	28%	*P* = .07
African American	38%	33%	39%	51%	*P* = .02
Age, mean (SD)	55.7 (8.5)	57.0 (8.3)	54.1 (7.4)	53.3 (9.3)	*P* = .002
Initial weight, mean (SD), kg	97.2 (16.2)	95.7 (16.1)	96.2 (15.7)	102.3 (16.2)	*P* = .009
Initial BMI, mean (SD)	34.2 (4.9)	33.5 (5.0)	34.2 (4.5)	36.2 (4.4)	*P* < .001
Attended > 15 of 20 Phase I sessions	88%	93%	85%	76%	*P* = .001
Phase I change in body weight, mean (SD)	-8.6 (4.5)	-8.9 (4.5)	-8.1 (4.3)	-8.4 (4.4)	*P* = .45
College or post college degree	62%	67%	61%	48%	*P* = .01
Annual income ≥ $60,000^b^	61%	61%	57%	63%	*P* = .78

^a^ Two-tailed *P* values based on Pearson chi-square test for categorical data and one-way ANOVA for continuous data

^b^ Missing values = 7; n for each group: all = 341, consistent = 208, some = 58, minimal = 75

**Table 2:** Overall use of specific website features over the 28 study months (N = 348)

Website Feature^a^	Median (Interquartile Range)
Log-ins	107 (52, 143)
Minutes spent on website	433 (236, 792)
Weight entries	104 (51, 130)
Exercise entries	124 (32, 376)
Sessions with added use after weight entry	86 (44, 116)
Bulletin board messages read	54 (14, 152)
Bulletin board messages posted	1 (0, 4)

^a^ Website features highly correlated with each other based on Spearman correlations

#### Consistent Use

Based on log-in and weight entry frequency described in the “Methods” section, 212 of 348 (61%) participants were defined as consistent users (Table 1). Relative to minimal users, consistent users were more likely to be older, male, other than African American, and more educated. Also, consistent users included participants who began Phase I at lower initial body weight and BMI and attended more Phase I weight-loss group sessions. With the exception of gender (*P* = .07), these differences were statistically significant. Participants in the “some use” group were generally intermediate to those in the “consistent use” and “minimal use” groups. No differences were observed between the website use categories for Phase I change in body weight or income.

**Figure 1:**
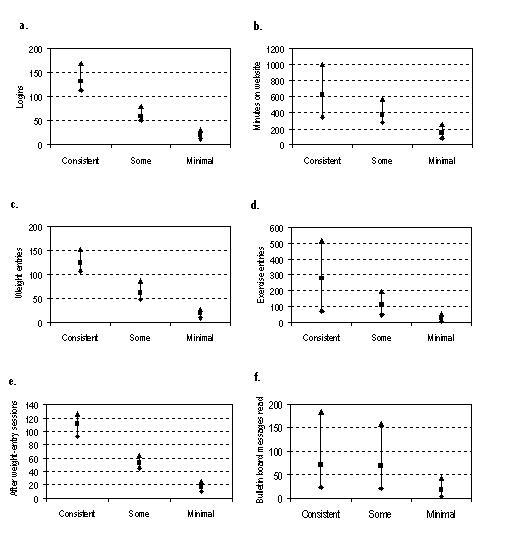
Use of various website features for consistent (n = 212), some (n = 61), and minimal (n = 75) use categories shown as median (square) with first (diamond) and third (triangle) quartiles

Figure 1 shows that consistent users engaged with all features of the website more often and spent more time on the website than other users. The website features were highly correlated with each other (Table 3).

**Table 3:** Spearman correlations of website use features

	Minutes on Website^a^	Weight Entries^a^	Exercise Entries^a^	After Weight-Entry Session^a^	Bulletin Board Messages Read^a^	Bulletin Board Messages Posted^a^
Log-ins	0.72	0.82	0.53	0.97	0.46	0.28
Minutes on website		0.68	0.65	0.66	0.56	0.35
Weight entries			0.56	0.85	0.34	0.17^b^
Exercise entries				0.53	0.23	0.12^c^
After weight-entry sessions					0.38	0.20
Bulletin Board messages read						0.64

^a^All values *P* < .001 unless otherwise specified

^b^ *P* = .001

^c^ *P* = .02

#### Website Use Associated with Weight Loss Maintenance

**Figure 2:**
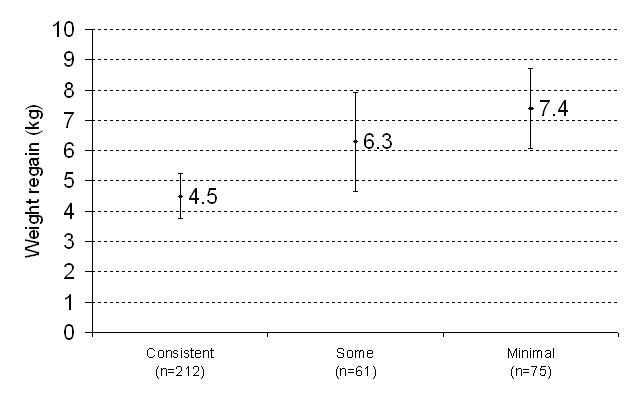
Phase II weight regain (kg) for each website use category shown as 95% confidence interval for the mean (two-tail *P* value testing for differences based on linear regression analysis adjusted for race, sex, education, income and age, *P* = .003)

**Figure 3:**
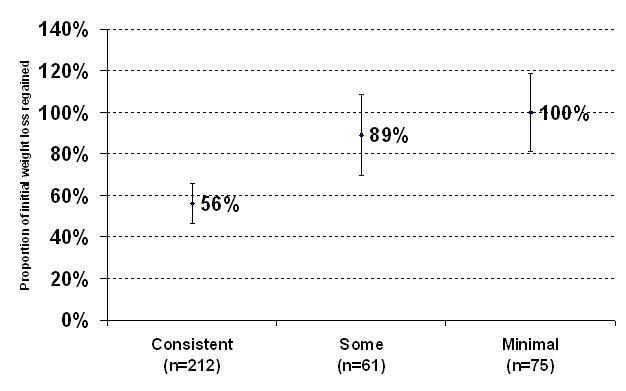
Proportion of initial weight loss regained for each website use category shown as 95% confidence interval for the mean (two-tail *P* value testing for any differences based on linear regression analysis adjusted for race, sex, education, income, and age, *P* = .001)

Figures 2 through 4 show the associations between website-use categories (consistent, some, and minimal) and three different weight outcome measures. Regardless of outcome measure, those in the consistent category had better weight outcomes compared with the other categories. Mean weight change in Phase II, both absolute weight and proportion of initial weight loss regained, was significantly lower (less weight regained) in the consistent website-use category (*P* = .003 [Figure 2] and *P* = .001 [Figure 3]). Likewise, significantly more consistent users, 107 out of 212 (51%), maintained a clinically important weight loss of 4 kg compared with 16 out of 61 (27%) and 18 out of 75 (24%) in the other two categories (*P* = .002). Although not significant (*P* = .45), a higher proportion of participants in the consistent category experienced no weight regain compared with the some and minimal categories, 34 out of 212 (16%), 6 out of 61 (10%), and 6 out of 75 (9%), respectfully*.* When additionally adjusted for initial BMI due to the differences between groups, the results do not change.

**Figure 4:**
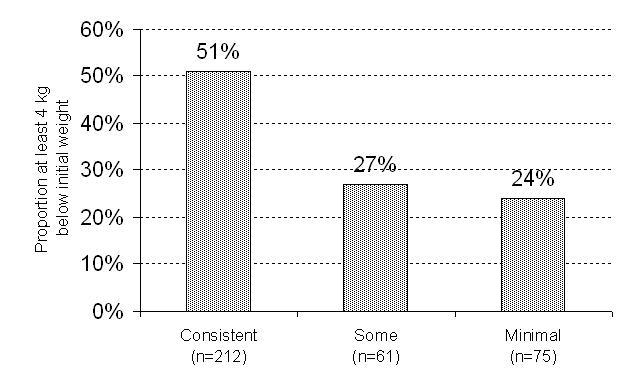
Proportion of participants at least 4 kg below initial weight for each website use category (two-tail *P* value testing for any differences based on logistic regression analysis adjusted for race, sex, education, income, and age, *P* = .002)

Table 4 shows top, middle, and bottom tertiles of use for specific website features and how frequency of use relates to weight loss maintenance. Greater numbers of log-ins, minutes, weight and exercise entries, and use of additional features after weight entry were associated with better weight outcomes for each weight outcome. Use of the bulletin board was associated with better weight outcome when that outcome was measured as either the proportion of initial weight regained or the proportion of participants at 4 kg below initial weight but was not significant when the outcome was measured as absolute weight regain. When the analysis was restricted to only consistent users (n = 212), the results were qualitatively similar (data not shown).

**Table 4:** Associations of various website features by tertile of use with different weight outcomes at end of data collection

		Top Tertile	Middle Tertile	Bottom Tertile	*P* value^a^
**Phase II weight change (regain) in kg, mean (SD)**					
	Log-ins	4.0 (10.1)	5.5 (9.4)	6.9 (9.6)	*P* = .001
	Minutes	3.1 (6.1)	6.2 (9.6)	6.9 (8.6)	*P* < .001
	Weight entries	4.8 (6.1)	4.3 (9.2)	7.2 (9.3)	*P* = .002
	Exercise entries	3.6 (10.5)	5.7 (9.3)	7.0 (8.8)	*P* < .001
	Added use after weight entry	4.3 (10.3)	4.9 (9.3)	7.1 (9.6)	*P* = .002
	Bulletin board posts	4.8 (13.2)	6.5 (10.2)	5.3 (7.1)	*P* = .10
	Bulletin board reads	4.7 (12.1)	6.2 (8.3)	5.4 (8.2)	*P* = .16
**Proportion of initial weight loss regained, mean (SD)**					
	Log-ins	48.8% (127.5%)	68.2% (98.9%)	96.4% (134.7%)	*P* < .001
	Minutes	36.3% (58.2%)	80.7% (137.5%)	95.7% (120.1%)	*P* < .001
	Weight entries	55.4% (73.0%)	56.4% (107.1%)	101.0% (121.9%)	*P* < .001
	Exercise entries	43.0% (108.6%)	78.5% (138.3%)	91.6% (113.5%)	*P* < .001
	Added use after weight entry	54.1% (132.0%)	59.8% (96.1%)	99.3% (137.5%)	*P* < .001
	Bulletin board posts	60.0% (159.0%)	88.4% (146.3%)	69.9% (90.9%)	*P* = .02
	Bulletin board reads	55.6% (136.0%)	82.1% (106.6%)	75.3% (115.2%)	*P* = .01
**Proportion of participants at least 4 kg below initial weight**					
	Log-ins	58.3%	39.5%	24.5%	*P* < .001
	Minutes	59.7%	36.9%	26.0%	*P* < .001
	Weight entries	49.1%	48.9%	24.6%	*P* = .005
	Exercise entries	61.2%	35.0%	26.2%	*P* < .001
	Added use after weight entry	54.8%	42.4%	25.2%	*P* = .002
	Bulletin board posts	47.4%	30.3%	41.6%	*P* = .05
	Bulletin board reads	53.9%	29.2%	39.5%	*P* < .001

^a^ Two-tail *P* values based on logistic (binary) or linear (continuous) regressions analysis adjusted for race, sex, education, income, and age

## Discussion

In this analysis, participants defined as consistent users of an interactive behavioral website designed to improve maintenance of weight loss had less long-term weight change (less regain). Consistent users were more likely to be older, other than African American and more educated, and used most features of the website more often when compared with other users. These results suggest that an Internet-based tool can provide some of the accountability and feedback assumed necessary for successful and long-term weight maintenance.

### Website Use

Previous studies using websites designed to improve weight loss have shown mixed results. Some research has shown that using a weight-loss website is strongly associated with short-term weight-loss success, but few long-term analyses are available, and even fewer consider the possible benefits of specific website features [8,18,19]. In a study of overweight and obese adults using an Internet-based weight-control program for 12 months, feedback website features (ie, progress charts) were the best predictors of initial 6-month weight loss, while social-support features (ie, Web chats and participant profiles) were related to weight maintenance at 12 months [8]. In a 6-month study, participants in an interactive Internet-based, behavioral weight-loss program lost significantly more weight than those who simply received access to the study’s website directory of selected weight-loss information [19]. Likewise, in a 12-month study, greater weight loss occurred in an Internet-plus-behavioral e-counseling intervention compared with those in just the basic Internet condition devoid of any email counseling [18]. On the other hand, no difference in weight regain was found at 12 months after a 4-month randomized behavioral weight-loss trial comparing a self-directed group with an Internet group that used a website with features like weight and exercise tracking, progress reports, and chat rooms [20]. This same study, however, found that in the Internet group use of the interactive diet-tracking features was related to weight change, a result similar to our findings.

Harvey-Berino et al [21] showed a clinically significant sustained weight loss 18 months after a 6-month behavioral weight-loss program for each of 3 intervention arms: frequent in person support, minimal in person support, and Internet-only support. The study concluded that the Internet was an effective vehicle for promoting clinically significant levels of weight loss, and that the Internet intervention was comparable to in person treatment up to 18 months. The WLM study showed promising weight-loss maintenance results for participants in the Internet group, compared with the self-directed control group at 18 months, but the effect was not sustained at 24 and 30 months [15]. This suggests that more research is needed to develop and promote a Web-based program that will produce sustainable long-term weight-loss maintenance.

### Internet Engagement

Keeping participants engaged in long-term treatment of weight management is the key challenge for any weight-loss program and is reflected in high study dropout rates. In a review of nine randomized controlled trials published from 1996 through 2002, mean study dropout rate was 21.2% at 18 months following completion of a 6-month treatment program [22]. In a study comparing three 12-month weight-maintenance programs, one of which was an interactive Internet intervention, after a 6-month behavioral weight-control program, 32% of those in the Internet condition did not complete the 18-month visit [21]. While dropout rates are a concern, reported Internet intervention participation rates are even more concerning [23,24]. In one study, only 30% of participants remained active after a 12-week physical activity intervention [24], while in another study, only 23% of participants visited a personalized Web-based cardiovascular risk reduction program at least once in the first 4 months of the study [25].

The low dropout rate and high long-term Internet participation rate of this study are likely due to several factors. The WLM website continually changed and presented a tailored welcome message and homepage with a “to do” list for each participant. The tailoring was a result of sophisticated programming that took into account the length of time since the participant’s last visit and features that the participant might benefit from based on the data entered. Our system of automated email and telephone prompts also may have contributed to high participation rates since 86% of all initial automated email prompts resulted in a subsequent log-in. Continued participation was also probably a result of initial individual training provided at randomization.

### Definition of Weight Loss Maintenance

Unfortunately, a standard definition for successful maintenance of weight loss does not exist [26]. We present our results using three different weight outcome measures to allow for thorough comparisons with other studies.

One way to define weight-loss maintenance is absolute weight change (regain) after initial weight loss. In this analysis, consistent website users regained less weight than those in other website-use categories. Also, those with higher counts of log-ins, minutes spent on the website, weight and exercise entries, and use after the weight entry regained less weight than those with lower counts. While absolute weight change during maintenance is an important outcome, it does not account for the total initial weight loss. Thus, the proportion of initial weight loss regained is a useful measurement. Using this second definition for weight-loss maintenance, the weight regain trend is similar to the absolute weight change results: consistent users regained 56% of initial weight lost compared with over 90% of initial weight regained among participants in the other categories.

We also present the proportion of participants whose weight is at least 4 kg below their initial weight. For adults, 4 kilograms of weight loss is generally accepted as clinically relevant [13,14]. This analysis demonstrated that significantly more consistent users and those in the highest tertiles of use for each website feature analyzed were more likely to be at least 4 kg below their initial weight.

This analysis has some limitations. Although, some subgroups in our population were more likely to be consistent users, our analysis did not include other factors that also may have contributed to being a consistent user. For instance, the screening process assured participants had regular access to the Internet; however, comfort level and familiarity with Internet-based tools, which were not assessed, also could have been a factor in consistency of use. This analysis did not assess the specifics of Internet access or how it may, or may not, have related to income and/or education. Internet access may have been from home, work, library, or somewhere else entirely, each of which may have some impact on consistency of use. Furthermore, the study design did not include integration of the interactive website into the initial Phase I weight-loss program. We speculate there may have been even greater use of the website in Phase II had this been done.

These observed Internet use associations do not prove causation. They are, however, consistent with our hypothesis that those who use a behaviorally based, interactive website more would be more successful at long-term weight maintenance. A major strength of this study is its long duration and large sample size, the diverse population (37% male, 38% African American), high follow-up data collection rate (93%), and high Internet participation rate (65% still logging on after 28 months) compared with other published Internet-use study results.

Participants defined as consistent users (based on regularity and frequency of use) of an interactive behavioral website designed to improve maintenance of weight loss had less long-term weight change (less regain), suggesting that Internet-based tools can provide some of the accountability and feedback assumed necessary for successful and long-term weight maintenance. The potential for widespread dissemination at relatively low cost per participant, even when the benefit is modest, makes further development of interactive-technology interventions worthwhile. Additional research is warranted of the relationship of long-term weight management with website-use patterns, use of specific behavior-change website features, and regularity of website usage.

## Abbreviations

BMI: body mass index
CVD: cardiovascular disease
NHLBI: National Heart, Lung, and Blood Institute
WLM: Weight Loss Maintenance Trial
